# Novel gene signatures for stage classification of the squamous cell carcinoma of the lung

**DOI:** 10.1038/s41598-021-83668-1

**Published:** 2021-03-01

**Authors:** Angel Juarez-Flores, Gabriel S. Zamudio, Marco V. José

**Affiliations:** Theoretical Biology Group, Instituto de Investigaciones Biomédicas, Universidad Nacional Autónoma de México, 04510 Ciudad Universitaria, Mexico

**Keywords:** Lung cancer, Small-cell lung cancer, Cancer, Biomarkers, Diseases, Oncology

## Abstract

The squamous cell carcinoma of the lung (SCLC) is one of the most common types of lung cancer. As GLOBOCAN reported in 2018, lung cancer was the first cause of death and new cases by cancer worldwide. Typically, diagnosis is made in the later stages of the disease with few treatment options available. The goal of this work was to find some key components underlying each stage of the disease, to help in the classification of tumor samples, and to increase the available options for experimental assays and molecular targets that could be used in treatment development. We employed two approaches. The first was based in the classic method of differential gene expression analysis, network analysis, and a novel concept known as network gatekeepers. The second approach was using machine learning algorithms. From our combined approach, we identified two sets of genes that could function as a signature to identify each stage of the cancer pathology. We also arrived at a network of 55 nodes, which according to their biological functions, they can be regarded as drivers in this cancer. Although biological experiments are necessary for their validation, we proposed that all these genes could be used for cancer development treatments.

## Introduction

As GLOBOCAN reported in 2018, lung cancer was the first cause of deaths and new cases by cancer worldwide^1^. Squamous cell carcinoma of the lung (SCC) is one type of lung cancer which comprises approximately 30% of all lung cancer cases. The available molecular targets for use in the treatment of SCC of the lung are behind of other types of cancer^2–4^. Recent advances in the treatment have been achieved using immunotherapy as nivolumab and pembrolizumab and some clinical trials are being conducted to test molecular targets^3,4^. Some efforts to understand the basis of the disease have been made using gene expression profiles, DNA sequencing and SNP arrays^2^. However, there are few preclinical murine models, some SCC of the lung cell lines have errors in their classification and molecular targets usually found in other types of lung cancer as lung adenocarcinoma are rarely present in SCC of the lung^4^. Lung cancer is classified into two wide groups as follows: Small cell lung cancer (SCLC) and non-small cell lung cancer (NSCLC). NSCLC represents 85% of all lung cancer cases. From this group the most prevalent are the adenocarcinoma and the squamous cell carcinoma of the lung^2,5^. Lung cancer survival is less than 5% after 5 years and most of them metastasize. Most of the time lung cancer is detected in advanced stages in which treatment is less effective. The best treatment is surgery although the effectivity of the treatment is linked to early stages of the disease^6–9^. Smoking is considered as a risk factor associated to lung cancer development^2^. Network analysis is widely used in different areas including biological sciences with a wide variety of results. There are different metrics that can be obtained from networks as the hubs which are commonly referred as the most connected nodes which lead to network instability if they are perturbed^10–13^. Besides, other network measures as betweenness and multivariate entropy have been used to analyze cancer networks to find putative potential targets for cancer disease^14,15^. We previously identified a set of nodes which due to its biological and network properties we called them network gatekeepers^16^. The latter was done by visual inspection. Gatekeepers have few nearest-neighbor interactions with other proteins, but these proteins have plenty of interactions. Gatekeepers might not be detected by standard differential gene expression analyses.

In this work, we use clustering centrality as a metric for a better and quicker identification of gatekeepers^16^. Machine learning algorithms have been applied to a wide variety of phenomena^17^. Health sciences have a special interest in the applications of these techniques due to the vast data publicly available with the objective to achieve better diagnosis and treatments of diseases. Some of the analyzed data with these approaches include analysis of histopathological images^18–20^. In this article, we make an analysis of the carcinogenic process of the squamous cell carcinoma of the lung using cutting-edge techniques as network and machine learning analyses to obtain sets of genes which could function as a signature to aid in the classification of patient tumor samples into one of the carcinogenic process stages and to increase the available options for experimental assays and molecular targets that could be used in treatment development. Although further biological experimental validation is needed.

## Results

### Carcinoma-stage classification model derived from machine learning

**Figure 1:**
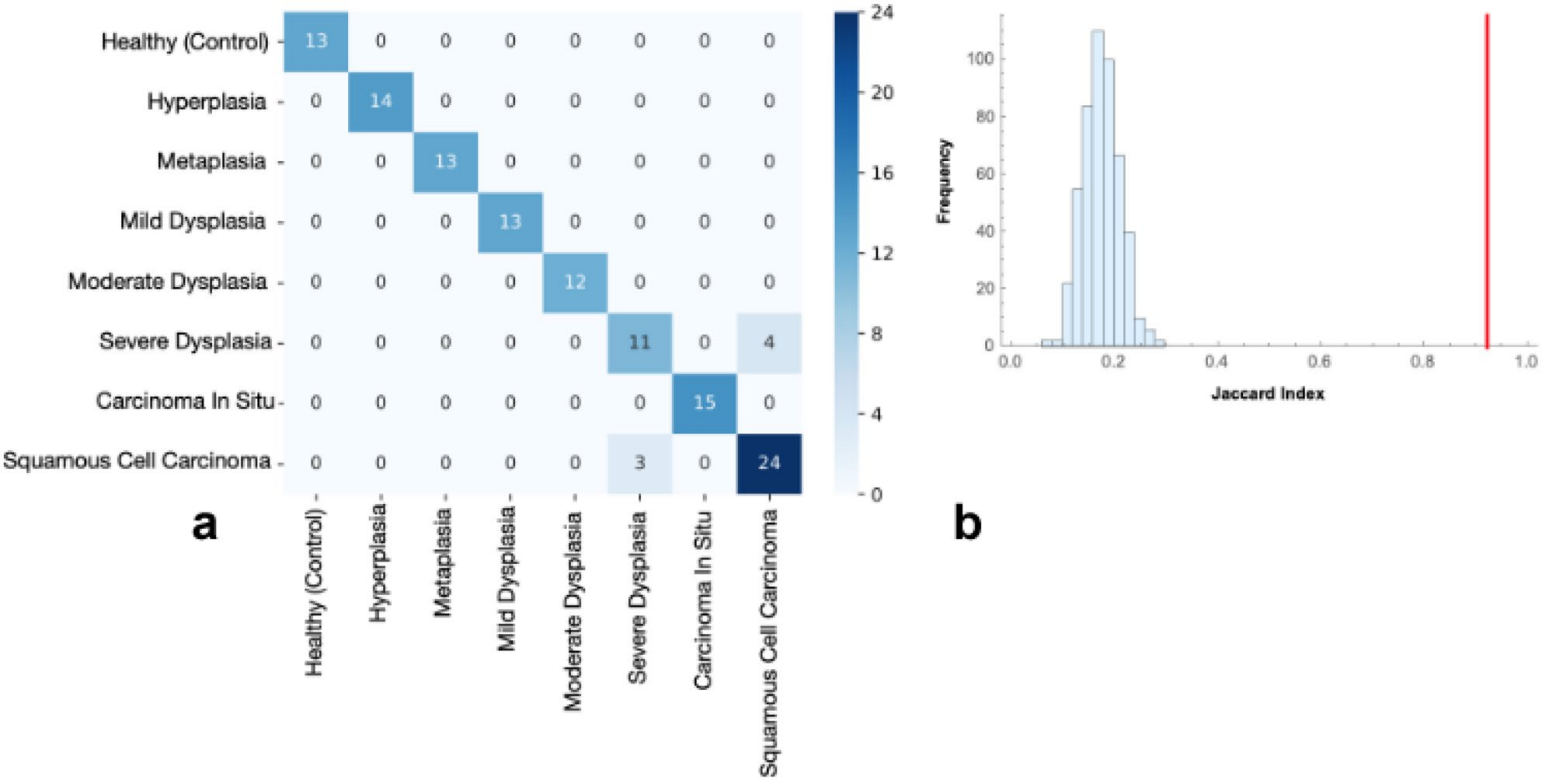
(**a**) Confussion matrix of the model trained with the 15 genes selected using the parameter reduction method. On the x-axis is the true classification and on the y-axis is the predicted classification for each of the 122 patient records. (**b**) Histogram of the Jaccard indexes from 500 trained models with random sets of 15 genes; In red the Jaccard index of the trained model with the 15 genes selected using the parameter reduction method. Figures were made using the library matplotlib of Python.

Data collected in GSE33479 was used to train a supervised machine learning algorithm. A logistic regression model was trained to classify the eight stages of the small cell lung carcinoma. Logistic regression models have been shown to provide accurate non-linear classification models of complex data^21^. A parameter reduction procedure was applied to the trained model. For this, the parameters of the model were standardized so that the parameters follow a standard normal distribution. The parameters whose value was beyond 0.78 from the mean were selected as relevant parameters. The procedure of model training and parameter selection was applied two times. On first parameter reduction, a total of 800 genes out of 41,067 were selected; on the second round a total of 15 relevant genes were selected. When using the subset of 800 genes a logistic regression model was trained and tested with records of all 122 patients records with all correctly classified, when considering the set of 15 genes the trained model was able to correctly classify the healthy stage and the first stages of small cell carcinoma and presented 7 cases of misclassification on later stages Fig. 1a. A neutral control was designed by considering a total of 500 random sets of 15 genes, on each random set a logistic regression model was trained, and its accuracy was measured using the Jaccard index and compared with the Jaccard index of a model trained with the selected set of 15 genes. The Jaccard index measures the proportion of correctly categorized cases by the model Fig. 1b. The Jaccard index from the set of genes derived from the parameter reduction method was of 0.92 whereas for the random sets the maximum Jaccard index was 0.29. When considering the set of 15 selected genes coupled with the 26 genes identified from previous analysis on PPI networks resulted in a trained model with a Jaccard index of 1. APID PPI data was used for network analysis of the results from the implemented machine learning technique for the first glance results of approximately 800 genes. APID was used due to better coverage of most part of the 800 genes.

### Differential gene expression analysis

**Figure 2:**
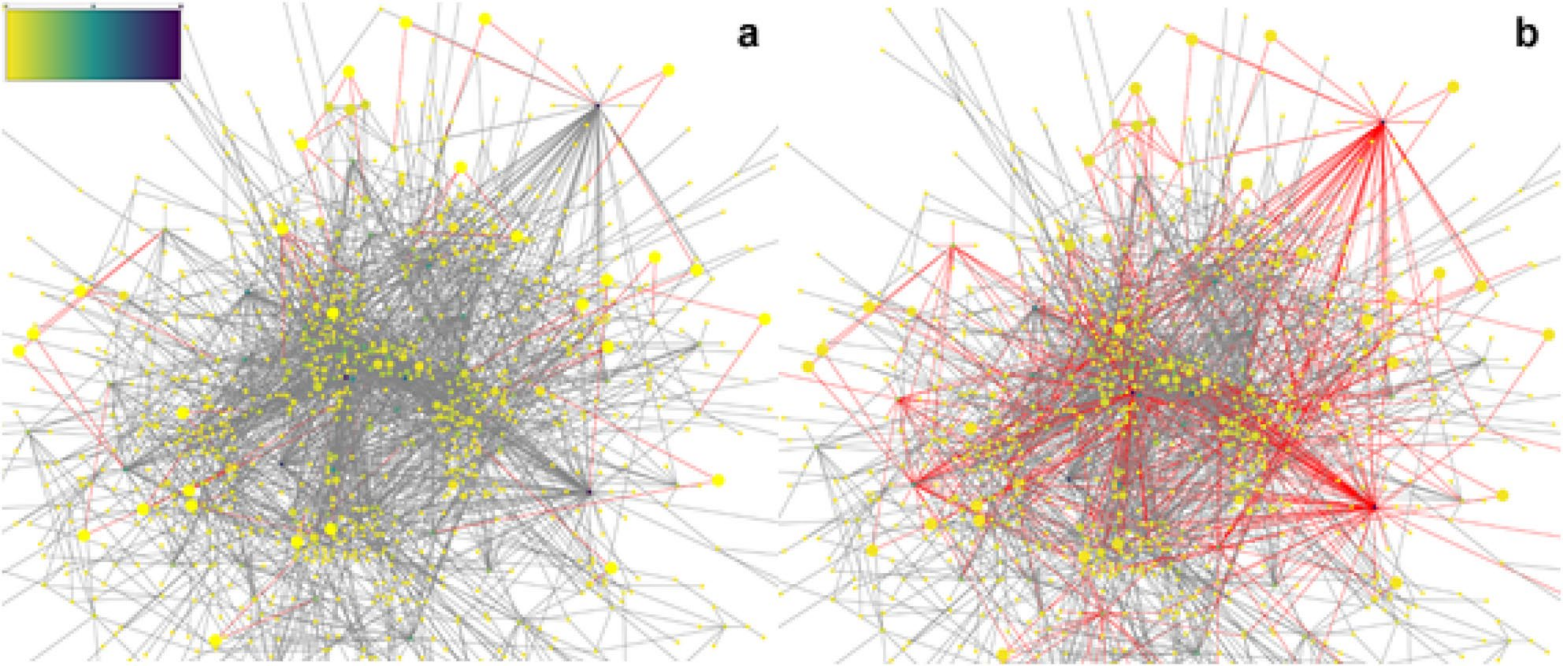
DGE-PPI network. (**a**) It represents a gathering and merging of the DGE analysis results and the Human Protein–Protein Interaction network from the Mentha Database. Red lines represent the connections of the gatekeepers. Color scale is presented in the left top corner. It represents the color scale applied to show graphically the values in the centrality measure for each node in the networks. (**b**) The red lines represent the connections of the gatekeepers plus the connections of its first neighbors (direct connected nodes to gatekeepers). The red lines comprise most of the network connections. (**a,b**) The proteins with less connections are marked in yellow and the most connected proteins are marked in navy blue purple. The size of each node represents the value of the clustering centrality measure; The bigger, the more value it has.

The first step was to carry out an exploratory network analysis which is shown in Figs. 2a,b. These networks are obtained from joining the results from the Differential Gene Expression (DGE) to Mentha network database and then the application of Eq. 1 to highlight the network gatekeepers. Figure 2a shows in the inset the color scale, which was applied in Fig. 2a,b. The minimum degree (number of connections of a node) value is 1 which is yellow in color, the most connected nodes are in navy blue purple whose value is 75. Figure 2a shows in red the connections that every gatekeeper has, and they are marked by bigger yellow circles. It can be observed that all of them are connected to other nodes, but they, at first glance, do not appear to be of importance because of the few connection they have.

In Fig. 2b can be observed in red, not only the gatekeeper’s connections but also the connections of the first connected nodes and how they have much more connections which comprises most of the network. The nodes at which gatekeepers are connected are hubs due to the highly connections they have.

**Figure 3:**
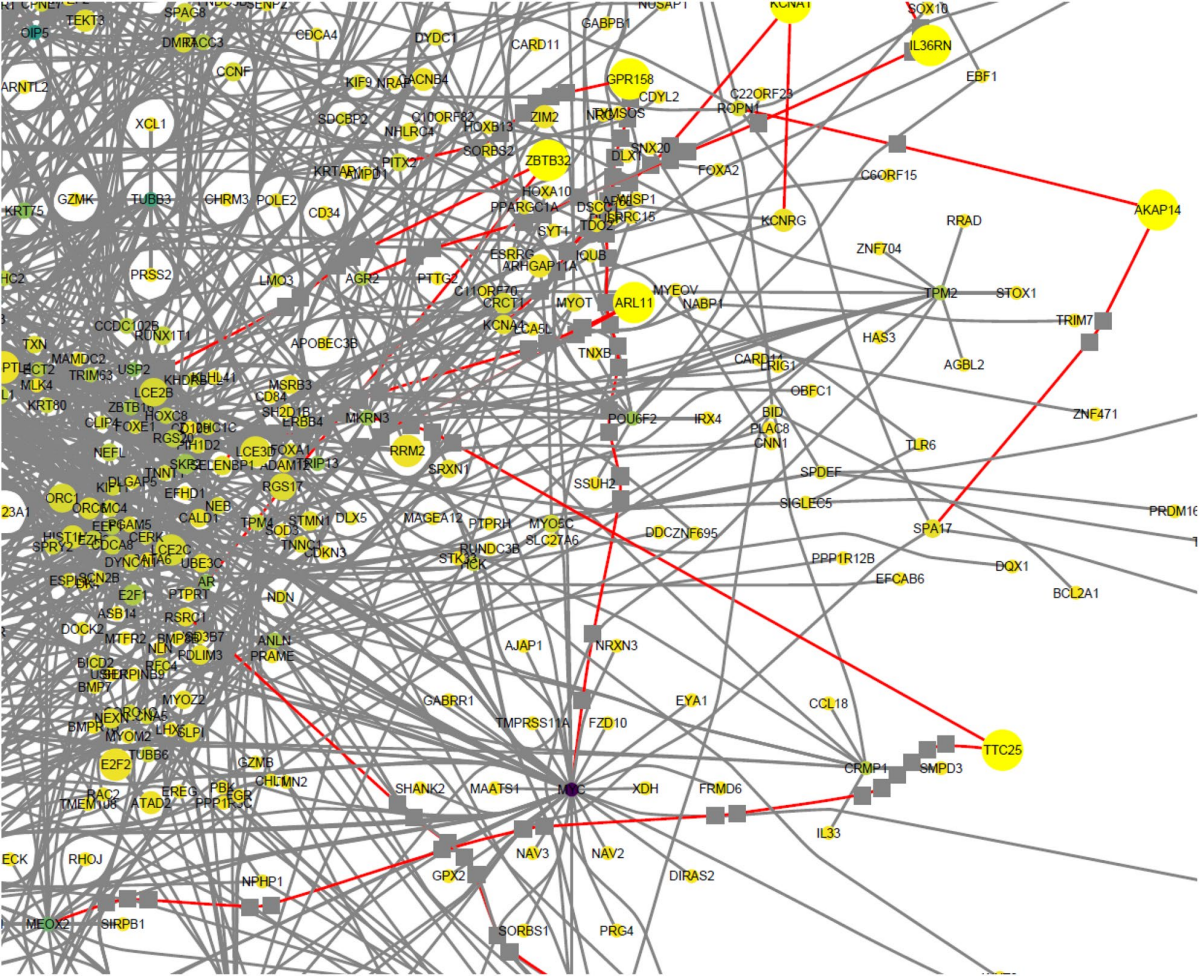
DGE-PPI network zoom. It can be observed with more detail some nodes with their respective connections. Red lines denote the connections of the network gatekeeper (nodes with a clustering centrality of 1) and some of the pointed nodes, in darker color, are associated to highly connected nodes.

In Fig. 3, a zoom of the graph of Fig. 2 is presented and each node is tagged with its HGNC name tag. It can be observed to which nodes some of the gatekeepers are connected. For example, a connection (red) to MYC protein (purple) can be observed. This protein is considered as an oncogene frequently associated with poor outcomes; a gatekeeper is linked to other proteins as MEOX2 whose possible function in some cancers is to be a suppressor gene^22,23^. Every one of the gatekeepers are linked to highly connected nodes which have relevant biological functions.

Table 1 summarizes in a list the results of the network analysis and the application of the machine learning algorithm in the GEO data set. A list of 26 gatekeepers’ proteins is displayed in the first column which were the proteins with a clustering centrality of 1 obtained by the network analysis. This set of genes were used as an input list for the machine learning algorithm in which the results showed that they can be used to identify each carcinogenic stage with great accuracy. Second and third columns are two lists that contain a reduced set obtained only from the machine learning algorithm to classify each sample into its corresponding stages. Second column contains the probe tag used by the chip. Using two different methods we obtained two list of potentially gene sets that could be used as an aid to help classify samples and whose biological functions denote their potential use as targets for therapy. Further experiments are needed to probe its potential use.

**Table 1:** Gene list from network gatekeepers and machine learning algorithm. Some Id are labeled as ** which means is a Missing Id. The first column corresponds to Gatekeepers list with 26 genes and the second column to the probe tag id in the microarray chip for 15 genes found with the machine learning method. The third column are the HGCN tags for each probe id of the second column. Second and third columns list finished when blank fields were present.

Gatekeepers (HGCN tags)	Probe tag Machine learning	HGCN tag for probe tag or genebank annotation
TTC25	A_23_P126803	ARPC5
SERPINA5	A_23_P216649	ABCA1
CENPL	A_23_P408865	Homo sapiens cDNA FLJ20700 fis, clone KAIA2250
ASF1B	A_23_P428366	HORMAD2
ZBTB32	A_23_P58009	C3orf52
GPR158	A_24_P100535	SYT15
RMI2	A_24_P141804	TMTC3
HSPB7	A_24_P239177	MUC4
ADRA1B	A_24_P515866	RBM6
GINS2	A_24_P542364	CALM1
APOC1	A_24_P59278	DSTYK
GINS1	A_24_P925678	PRG2
CENPK	A_24_P937366	**
KCNA1	A_32_P213091	LOC440338
PI3	A_32_P429083	LOC441621
ATP6V0D2		
ALS2CR12		
IL36RN		
KIF26B		
SPC25		
ARL11		
UBXN10		
LUM		
COTL1		
RYR3		
CENPI		

An enrichment test was performed using the gatekeepers list to discover the main pathways associated with them as shown Table 2. The first characteristic is that every category is overrepresented, which means that in each presented category there are more genes from the input list than it can be expected (using as reference the *Homo sapiens* REFLIST) and most of the processes are involved in cell cycle-related specially in mitosis.

**Table 2:** Gatekeepers: Enrichment test-Reactome pathways. Main reactome pathways are shown if False discovery rate value was less than 0.05.

Reactome pathways	Homo sapiens—REFLIST (20,851)	Client Text Box Input (27)	Client Text Box Input (over/under)	Client Text Box Input (FDR)
Unwinding of DNA (R-HSA-176974)	12	2	+	2.07E−02
Deposition of new CENPA-containing nucleosomes at the centromere (R-HSA-606279)	54	3	+	1.23E−02
Nucleosome assembly (R-HSA-774815)	54	3	+	1.12E−02
Amplification of signal from unattached kinetochores via a MAD2 inhibitory signal (R-HSA-141444)	92	4	+	1.55E−02
Amplification of signal from the kinetochores (R-HSA-141424)	92	4	+	7.76E−03
Mitotic Spindle Checkpoint (R-HSA-69618)	108	4	+	7.15E−03
EML4 and NUDC in mitotic spindle formation (R-HSA-9648025)	114	4	+	7.03E−03
Chromosome Maintenance (R-HSA-73886)	90	3	+	3.11E−02
Resolution of Sister Chromatid Cohesion (R-HSA-2500257)	122	4	+	7.59E−03
RHO GTPases Activate Formins (R-HSA-5663220)	135	4	+	8.37E−03
Separation of Sister Chromatids (R-HSA-2467813)	185	4	+	1.85E−02
Mitotic Anaphase (R-HSA-68882)	193	4	+	2.00E−02
Mitotic Metaphase and Anaphase (R-HSA-2555396)	194	4	+	1.89E−02
Mitotic Prometaphase (R-HSA-68877)	198	4	+	1.91E−02
Cell Cycle Checkpoints (R-HSA-69620)	270	5	+	7.94E−03
Cell Cycle, Mitotic (R-HSA-69278)	495	6	+	9.07E−03
Cell Cycle (R-HSA-1640170)	600	7	+	6.91E−03

The next step was to search in distinct databases the list of genes obtained by the machine learning algorithm. We selected two pathway databases: the reactome pathways and the KEGG pathways. In Table 3, it can be observed a list of 8 genes for which information was available. The first column corresponds to its name, the second column to the related pathways in Reactome and the third to KEGG pathways. Some of the related pathways are usually altered in some types of cancer as Beta-catenin independent WNT-signaling, SMAD2/SMAD3, tight junction, ABC transporters, etc.^24–27^.

**Table 3:** Machine learning selected genes: Reactome and KEGG pathways involved. The pathways that could be related to squamous cell carcinoma of the lung are shown in Reactome column. Most of the genes do not have a pathway related to KEGG database, they are labeled as null. If there were more than three pathways available in either database just three pathways or less were selected when its biological function could be useful in cancer progression, growth, or maintenance. If just one pathway was available, it was written in the corresponding field. Null is used when no hits were found in the database. Only. genes that do not appear in either database were not presented.

Name	Reactome	KEGG
ARPC5	EPH-Ephrin signaling	Tight junction, Regulation of actin cytoskeleton, Bacterial invasion of epithelial cells
ABCA1	Regulation of lipid metabolism by PPARalpha	ABC transporters
HORMAD2	Recruitment and ATM-mediated phosphorylation of repair and signaling proteins at DNA double strand breaks Processing of DNA double-strand break ends Nonhomologous End-Joining (NHEJ)	Null
C3orf52	SMAD2/SMAD3:SMAD4 heterotrimer regulates transcription Complex I biogenesis	Null
TMTC3	Reelin signalling pathway	Null
MUC4	O-linked glycosylation	Null
CALM1	Beta-catenin independent WNT signaling RAS processing RAF/MAP kinase cascade Signaling downstream of RAS mutant Signaling by RAF1 mutants	Null
PRG2	Neutrophil degranulation	Asthma

**Figure 4:**
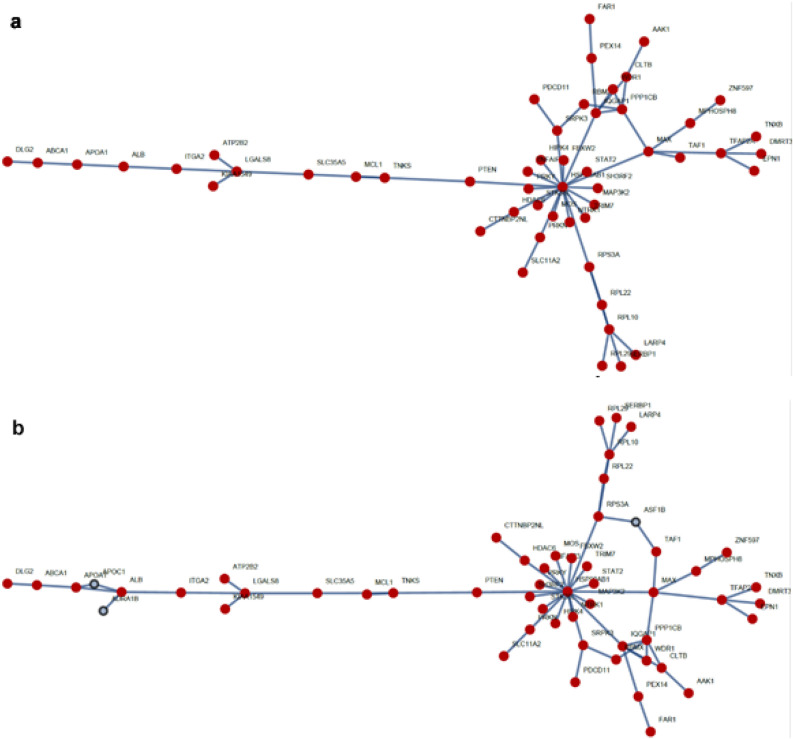
Big component network. (**a**) A network with 52 nodes is displayed. The network is a big component observed in the exploratory network analysis of a network created by the Machine learning algorithm. Nodes are displayed in red color; connections are in blue. (**b**) A network of 55 nodes of which 3 nodes are from the identified gatekeepers. Figures were made with the library NetWorkX of Python.

A network was made based in the results obtained from the machine learning algorithm first glance which comprised approximately 800 genes. It was observed a big component (when a significant proportion of the nodes in a graph are connected) created by some nodes as seen in Fig. 4a.

An interesting characteristic of the identified network by the machine learning algorithm was that some of the network gatekeepers identified by the DGE analysis were connected to this big component as seen in Fig. 4b. Some biological functions of some nodes in this network are well known to be relevant for cancer progression as PTEN which is a tumor suppressor altered in some types of cancer, as well as others like MCL1 which is an anti-apoptotic protein altered in some types of cancers. Also, MCL1 is being studied as a target for cancer patient treatment in small cell lung cancer^28,29^. FAR1 is observed to play an essential role in the production of ether lipids/plasmalogens whose synthesis requires fatty alcohol. ABCA1 catalyzes the translocation of specific phospholipids from the cytoplasmic to the extracellular/luminal leaflet of membrane coupled to the hydrolysis of ATP. In cancer it was observed that its inhibition plays an important role for cancer survival due to an increase of mitochondrial cholesterol. The function of DMRT3 is not clear. It is thought to function as a transcription factor. In a study of lung cancer, the dysregulations of DMRT3 along with other two proteins were considered specific for lung squamous cell carcinoma ^30^. AAK1 is a kinase that participates in the regulation of clathrin-mediated endocytosis. It was discovered that in β-Catenin-dependent WNT signal a negative feedback loop is created by its expression. ASF1B Is a histone chaperone which facilitates histone deposition, exchange, and removal during nucleosome assembly/disassembly; in cervical cancer it was observed that it functions as an oncogene accelerating cancer cells proliferation. APOC1 functions as an inhibitor of lipoprotein binding to the low-density lipoprotein (LDL) receptor. In gastric cancer it was proposed as a potential diagnostic and prognostic biomarker; in colorectal cancer evidence points out to have a promoting role in carcinogenesis. ADRA1B is an alpha-adrenergic receptor whose action is mediated by association with G proteins; in gastric cancer it was found a methylation promoter and it could be frequently involved in development and gastric cancer progression. These mentioned proteins are other examples of gene protein products whose functions are or could be involved with cancer disease^30–37^.

## Discussion

Lung cancer is the deadliest type of cancer, most of the diagnosed cases are made in the last stages of the disease and there are little available treatment options which could have an important effect. Small cell carcinoma of the lung comprehends a great part of all lung cancers. Our present results provide a better comprehension of the underlying components of the disease. The detection of genes and proteins that could be implicated in the carcinogenic process is urgently needed to provide better options for treatments and diagnosis. Herein, we performed a thorough search of genes and proteins that could be used to offer better treatments and diagnosis options. We made a comprehensive analysis of all the carcinogenic process and observed that some set of genes could be used as an aid for small cell carcinoma of the lung stage classification. We employed two pathways to identify relevant genes for diagnosis. The first was based in a classic method as DGE analysis with the aid of more recent techniques as network analysis with a novel concept as the network gatekeepers which are encountered by using clustering centrality. DGE analysis was used as an exploratory analysis to look for possibly patterns in the gene expression for each stage. Although a general panorama of the carcinogenic process was obtained, we wanted to summarize it into a small meaningful set of genes with high involvement in cancer development. To make this possible we used the output data of the DGE as the input for the network analysis and then search for the network gatekeepers. The other pathway was based in another cutting-edge technique, machine learning algorithms. Hitherto, machine learning applications on cancer have been for assessing cancer prediction and prognosis^38^. These results are based on the analysis of a wide set of variables including biomarkers and clinical factors such as age, location and type of cancer, and size of tumor^39–41^. The results presented in here are not intended for cancer prevention or survivability directly, rather they provide a set of specific genetic biomarkers whose analysis can lead to an immediate diagnosis about the stage of development of small cell carcinoma in a patient. The analysis of the proposed genetic biomarkers can differentiate even the earliest stages of cancer development and lead a physician to administer the required treatment when the probabilities of survivability of the patient are higher. For each method we found a set of genes which we proposed to be useful as an aid for stage classification and due to the important biological roles in which they are involved they could also be useful for further validation as possible targets for treatment. The biological roles of the gatekeepers proposed set are marked as cell cycle regulation, DNA-repair breaks, nucleosome assembly and processes that occur in the mitotic phase. It was first observed in the gatekeeper network that most of them exhibit scarce connections but their first neighbor nodes which are directly connected are hubs (highly connected nodes). This along with the processes they are involved may permit an access for prior processes regulated by the hubs. It is known that network hubs are of high importance for network stability, but in this work, we are observing that it can be of great importance to use the network gatekeepers as a measure to find key components in a biological context. The machine learning algorithms are usually used in other fields to improve the understanding of a wide variety of processes. In the case of cancer its aim is to find new targets and possible key proteins that regulate cancer. We found a reduced set of genes that can be used for stage classification in a set of microarray data and this also can be done with the set of gatekeepers. The biological functions of each of the identified genes are relevant for normal stages and as previously observed for cancer development. For example, in the case of the reduced set obtained with machine learning, ARPC5 is a protein whose normal function is involved in EPH-Ephrin signaling and tight junction regulation and they are involved in cancer processes as adhesion, migrations, invasion or growth. This protein was recently proposed to be a prognostic biomarker for patients with multiple myeloma^42^. In the case of ABCA1 is a protein whose inhibition promotes cancer progression^32^. Genes identified in the big component obtained from the machine learning first set was also analyzed and observed that some of them were previously studied in cancer and that their functions are involved with them. It is necessary to study these proteins in the context of squamous cell carcinoma of the lung, as it is known that the function is dependent of the type of tissue, microenvironment, and type of cancer. Our combined approach of DGE analysis plus the use of the metric of clustering centrality together with the application of machine learning algorithms, will facilitate the identification of relevant components in biological networks as the ones derived from cancer data.

## Conclusions

We found a small set of genes possibly involved in the development of the disease. We propose two sets of genes which could help in the classification of tumor samples. These findings can increase the available options for experimental assays and molecular targets that could be used in novel treatment development. Although further experimental research is needed to validate their utility in the clinical setting.

## Methods

**Figure 5:**
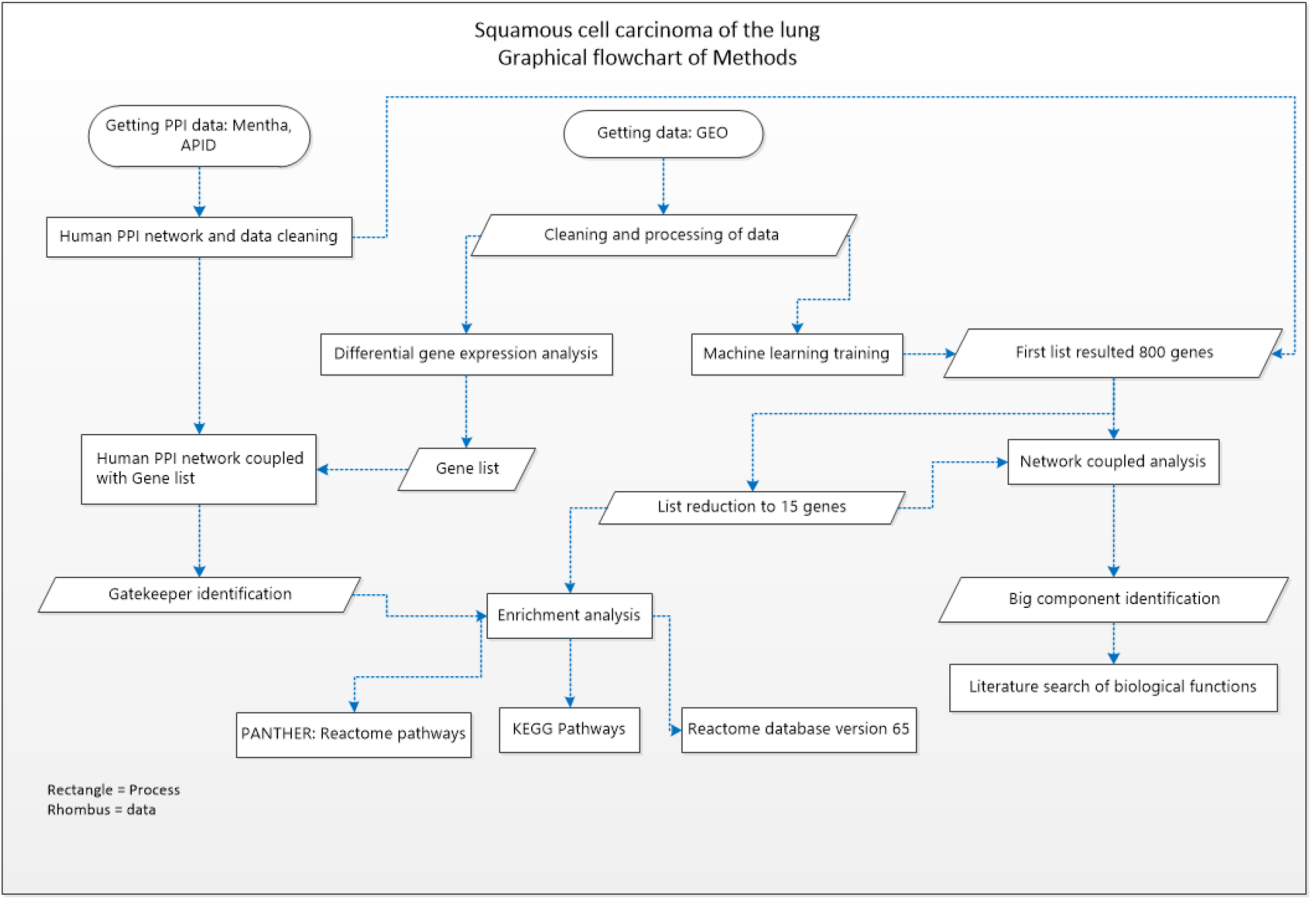
Workflow. A general panorama of the methodology and the databases. Figure made with Microsoft Visio.

A graphical flowchart that summarizes the methods and the data bases is shown in Fig. 5. Data collection was made by using various databases: Gene expression Omnibus for gene expression patients set, GEO accession: GSE33479, which comprises 122 patient samples representing the carcinogenic stages. Samples were divided as: 13 normal histology and normo-fluorescent, 14 with normal histology and hypo-fluorescent, those were grouped as the control group, 15 metaplasia samples, 13 mild dysplasia, 13 moderate dysplasia, 12 severe dysplasia, 13 carcinoma in situ, and 14 for squamous cell carcinoma of the lung. The gene expression platform was Agilent-014850 Whole Human Genome, Microarray 4 × 44 K G4112F. Processing and differential gene expression analysis were performed using R v3.5.1 software (http://www.R-project.org). Processed data retrieval was performed by GEOquery R package. Hgug4112a.db R package was used to annotate each gene ID to the data^43^. Using limma package differential gene expression (DGE) analysis was used to compare each stage vs the normal. Limma package fits a generalized linear model before comparisons and then calculate a moderate t-statistic for each contrast^44,45^. A p-value is obtained which is adjusted based in Benjamini and Hochberg False Discovery Rate correction^44,46^. A list from the DGE was obtained for each comparison, results were merged to obtain a new list with all differentially genes. Full Human Interactome was downloaded from Mentha and APID database^47,48^. Protein–protein interactions (PPI) level 0 data (all reported proteins pairs) was obtained from APID. Cleaning process for both networks was made using Cytoscape software (Networks for Figs. 2 and 3 were created using this software) which comprised: deletion of repeated interactions, deletion of protein interactions detected in other organism, deletion of self-loop interactions in proteins^49^. Both databases are public and free to use. The merged list resulted from DGE (using a filter of p < 0.05 and Fold change < − 1.5 & > 1.5) from the microarray data were coupled with Mentha PPI dataset which allowed to create a new network of PPIs which were used as a template to identify the network gatekeeper’s proteins using clustering centrality measure Eq. (1). Mentha PPI data was used due to the better coverage of the genes that appeared in list of DGE analysis. To calculate clustering centrality measure we used the following Eq. (1):

$$C_{i} = \frac{{2E_{i} }}{{k_{i} (k_{i} - 1)}}$$

where *C*_*i*_ is the clustering coefficient of a node *i* and is defined as the fraction *E*_*i*_ of existing connections among its *k*_*i*_ nearest neighbors divided by the total number of possible connections.

### Enrichment test

Statistical overrepresentation analysis was performed using PANTHER database for Reactome Pathways applied to Gatekeepers list using Fisher exact test. Raw *p* values were obtained. This value is the probability that the number of observed genes in each category occurred by chance. These p-values were corrected using False Discovery Rate by Benjamini-Hochberg. The reference list used was for *Homo sapiens*. Reactome database version 65 Released 2019-12-22 was used. In the case of machine learning gene list, it was not possible to use the PANTHER database due to the lack of information about them, none of them was annotated in the database, instead Reactome and KEGG Pathways database were used to perform individual searches of each gene in the list^50–54^.

## Data Availability

The datasets generated analyzed during the current study are available in the GEO, Mentha and APID repository. GEO: https://www.ncbi.nlm.nih.gov/geo/query/acc.cgi?acc=GSE33479 Mentha for human: https://mentha.uniroma2.it/ APID for human: http://cicblade.dep.usal.es:8080/APID/init.action. The datasets generated during the current study are available from the corresponding author on reasonable request.
